# Association of lung immune prognostic index with overall survival in pancreatic ductal adenocarcinoma patients treated using chemotherapy

**DOI:** 10.7150/ijms.102404

**Published:** 2025-03-03

**Authors:** Nan Zhang, Guochao Deng, Ru Jia, Quanli Han, Guanghai Dai

**Affiliations:** 1 Medical School of Chinese PLA, Beijing 100853, China.; 2 Department of Medical Oncology, the Fifth Medical Center, Chinese PLA General Hospital, Beijing 100071, China.; 3 Department of Medical Oncology, the First Medical Center, Chinese PLA General Hospital, Beijing 100853, China.

**Keywords:** pancreatic ductal adenocarcinoma, Lung Immune Prognostic Index score, overall survival

## Abstract

**Background:** The lung immune prognostic index (LIPI) has attracted considerable interest for its prognostic value in several malignancies. However, its prognostic value in pancreatic ductal adenocarcinoma (PDAC) has not yet been clarified.

**Objective:** This study aimed to assess the role of LIPI with regard to overall survival (OS) in locally advanced or metastatic PDAC patients undergoing chemotherapy.

**Methods:** Data from 256 patients with PDAC treated via chemotherapy at the Chinese PLA General Hospital between January 1, 2011 and July 1, 2018 were retrospectively reviewed. Their neutrophil-to-lymphocyte ratio (dNLR) with lactate dehydrogenase (LDH) values were used to calculate each one's LIPI. The Cox proportional hazard model was used to identify the association between LIPI and OS.

**Results:** Of the included patients, 154 were in the good LIPI group and 102 were in the intermediate/poor LIPI group. The OS in the two groups were 9.0 months (95% CI: 7.351-10.649) and 6.0 months (95% CI: 4.812-7.188), respectively. Patients in the good LIPI group had better OS compared to those in the intermediate/poor LIPI group (HR, 0.720; 95% CI: 0.554-0.935; *P* = 0.014).

**Conclusion:** This study revealed LIPI is significantly associated with OS in PDAC and could play a significant role in helping clinicians make appropriate decisions for PDAC patients undergoing chemotherapy.

## Introduction

Pancreatic ductal adenocarcinoma (PDAC) is among the deadliest and highly metastatic forms of cancer, with a projected 5-year survival rate of merely 3% 1, 2. There are several factors that contribute to its invasiveness, such as the absence of early diagnostic markers, delayed detection caused by the lack of symptoms, intricate genetic characteristics, and early spread of metastasis 3, 4. Complete surgical removal is currently the sole potentially curative approach for patients diagnosed with metastatic PDAC, with the potential to increase the 5-year survival rate to around 20% 3, 5, 6. However, > 80% of patients with PDAC have unresectable tumors at their time of diagnosis, most often due to vascular invasion and distant metastasis 7. According to the ESMO guidelines, chemotherapy remains the primary treatment for pancreatic cancer 3. With medical advancements, some patients with pancreatic cancer have achieved survival of more than one year. However, there are still patients with limited sensitivity to cytotoxic drugs who succumb to the disease due to ineffective treatment 8. Therefore, it is particularly important to identify biomarkers that can effectively predict the prognosis of PDAC. Such biomarkers would help doctors to early identify patients who may not respond well to conventional chemotherapy. This, in turn, would allow for the optimization of treatment strategies and the exploration of additional treatment options, ultimately improving survival rates and quality of life 9.

The derived neutrophil-to-lymphocyte ratio (dNLR) reflects the composition of the tumor microenvironment, which determines the tumor's ability to evade the immune system 10. Lactate dehydrogenase (LDH) plays a crucial role in the final step of glycolysis, providing both energy and biosynthesis precursors to tumor cells. Its impact on tumor survival is primarily through the inhibition of apoptosis, prevention of necrosis in hypoxic environments, and protection from damage caused by reactive oxygen species 11. The lung immune prognostic index (LIPI) a compositional biomarker, was developed to reflect the association between dNLR and the blood LDH levels. LIPI was first reported by Mezquita et al., who found it to be significantly associated with the systemic inflammatory response and prognosis of non-small cell lung cancer following treatment with immune checkpoint inhibitors 12. Increasing attention has been paid to LIPI in the field of extrapulmonary tumors 13, 14. It has been shown to be significantly related to the prognosis of various cancers, including: osteosarcoma patients receiving standard treatment 15, esophageal squamous cell carcinoma patients undergoing radical surgery or chemoradiotherapy 13, 14, urothelial bladder cancer patients undergoing radical cystectomy 16, and advanced breast cancer patients receiving trastuzumab therapy 17. This growing body of evidence highlights the potential of LIPI as a valuable prognostic tool across different types of cancer.

The short overall survival (OS) of patients with PDAC is not only related to the disease stage, but also to the fact that the current treatment mainly relies on chemotherapy 18. However, chemotherapeutic drugs face challenges when entering the internal environments of PDAC tumors 19. The unique immunosuppressive microenvironment and dense stromal matrix of PDAC contribute to the low efficacy and short survival times associated with chemotherapy 20, 21. To data, no studies have identified the potential roles that LIPI might play in predicting the prognosis of PDAC in patients undergoing chemotherapy. Thus, this study evaluated the prognostic role of LIPI regarding overall survival in patients with PDAC undergoing chemotherapy.

## Participants and Methods

### Study population

A retrospective review was conducted on a cohort of 256 patients with PDAC who received treatment at the Chinese PLA General Hospital from January 1, 2011 to July 1, 2018. Inclusion criteria for patients were as follows: (1) adults diagnosed with stage IV or locally advanced PDAC, (2) treated via chemotherapy, and (3) laboratory examinations were performed within one week before the initiation of treatment. Patients were excluded if: (1) laboratory examination results were not obtained; (2) patients underwent radical resection; and (3) patients with malignancies in other organs, inflammatory conditions, autoimmune disorders, or injuries. Informed consent was waived by the committee because of the retrospective nature of the study. This study was approved by the Ethics Committee of the PLA General Hospital (ethical approval number: S2014-031-01). Clinical data were electronically retrieved from the medical records of the PLA General Hospital Registry. All treatments were performed in accordance with the institution's guidelines and regulations.

### Recorded variables

Demographic and clinical variables were obtained from the patients' electronic medical records. Which included age, sex, smoking status, alcohol status, carcinoembryonic antigen (CEA), carbohydrate antigen 19-9 (CA19-9), LDH, platelets (PLT), albumin (ALB), diabetes, obstructive jaundice, chest/abdominal effusion, history of organ transplantations, liver metastases, and chemotherapy regimens. The chemotherapy regimens used included: (1) the GS regimen: S-1 (40-60 mg, twice daily, given orally after breakfast and dinner for 14 days, followed by 7 days off), and gemcitabine (1,000 mg/m^2^ intravenously given on the first and eighth days of each cycle); (2) the AS regimen: S-1 (40-60 mg, twice daily, given orally after breakfast and dinner for 14 days, followed by 7 days off), and paclitaxel (260 mg/m^2^ intravenously given on the first and eighth days of each cycle); and (3) the GEMOX regimen: oxaliplatin (130 mg/m^2^, given intravenously on the first day) combined with gemcitabine (1,000 mg/m^2^, given intravenously on the first day), 14 days per cycle. Treatment regimen selection was based on each patient's pathological stage, general health condition, and other considerations.

### LIPI definition and grouping

LIPI is based on LDH and dNLR. We used dNLR before treatment as the variable, and a normal LDH level was defined as 0-250 U/L. Survival receiving operator characteristic (ROC) curves were used to calculate dNLR in order to predict OS at 2.3, using X-tile software (Version 3.6.1). Patients were divided into three groups based on their dNLR (above the optimal cutoff value) and LDH levels (above the upper limit of normal): good (total score of 0), intermediate (total score of 1), and poor (total score of 2).

### Outcome definition

The outcome investigated was overall survival (OS), defined as the time from the beginning of chemotherapy until death.

### Statistical analysis

The demographics and characteristics of the good and intermediate/poor LIPI groups were assigned as categorical and continuous data, respectively. Categorical data are presented as frequencies and percentages, while continuous data are reported as means (with standard deviations) or medians (with ranges) depending on the distribution of the data. Chi-squared or Fisher's exact tests were used to compare the differences between groups. The optimal cutoff value of dNLR was evaluated using ROC curves, and the survival analysis was performed using the survival curve and Kaplan-Meier analysis. Cox proportional hazards regression analysis was employed to evaluate the prognostic significance of the LIPI for OS, and hazard ratios (HRs) with corresponding 95% confidence intervals (CIs) were computed as the measure of effect. Stratified analyses were performed to assess the prognostic role of LIPI for OS according to patients' characteristics. All P values reported in this study are two-tailed, and the significance level was set at 0.05. Statistical analyses were conducted using SPSS 26.0 software (Chicago, IL, United States).

## Results

### Baseline patient and group characteristics

A total of 256 patients (154 male and 102 female) were enrolled, with a mean age of 55.46 years. Forty-three had locally advanced PDAC with no chance for surgical treatment, and the remaining 213 had metastatic PDAC. Of those with metastatic PDAC, 194 had liver metastasis and 54 had ≥ 2 metastases. Each patient's treatment plan was based on their ECOG score, tumor stage, and general condition.

**Figure 1:**
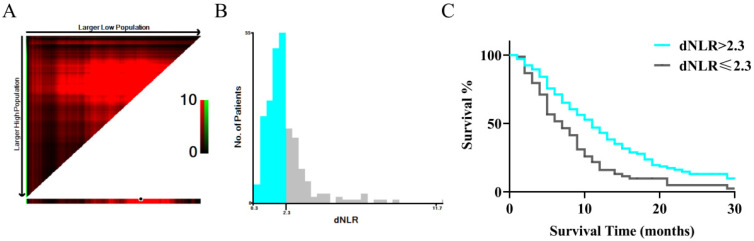
ROC curve of pretreatment dNLR in assessment of the overall survival at 2.3 through X-tile Software. In figure 1A, the X-axis represents all potential cut-points from low to high (left to right) that define a low subset, whereas the Y-axis represents cut-points from high to low (top to bottom), that define a high subset. The arrows represent the direction in which the low subset (X-axis) and the high subset (Y-axis) increase in size. Red coloration of cut-points indicates an inverse correlation with survival, whereas green coloration represents direct associations. The optimal cut-point occurs at the brightest pixel (green or red). The optimal cut-off point is shown on a histogram of the entire cohort in figure 1 B. In figure 1C, a Kaplan-Meier curve is plotted to show the correlation of dNLR with OS.

Using dNLR as the independent variable, and based on the time-dependent ROC curve (Figure 1), the optimal cutoff value was determined to be 2.3. LDH levels within the range of 0-250 U/L were considered normal. The patients were divided into three groups based on their LIPI scores: the good group (total score of 0) consisted of 154 patients, the intermediate group (total score of 1) consisted of 89 patients, and the poor group (total score of 2) had 13 patients. Owing to the limited number of patients in the poor group, the intermediate group was merged with the poor group. A total of 154 (60.2%) patients had good LIPI scores, while 102 (40.9%) had intermediate/poor LIPI scores.

The demographic characteristics of the participants in both groups are presented in Table 1. There were notable disparities observed between the groups in relation to CEA (*P* = 0.006), LDH (*P* < 0.001), dNLR (*P* < 0.001), obstructive jaundice (*P* = 0.003), chest abdominal effusion (*P* = 0.041), and number of organ transplants (*P* = 0.019). We also observed no significant differences in age, sex, smoking status, alcohol consumption, PLT, ALB, diabetes, liver metastases, or chemotherapy regimens between the groups.

### The prognostic role of LIPI on OS

**Figure 2:**
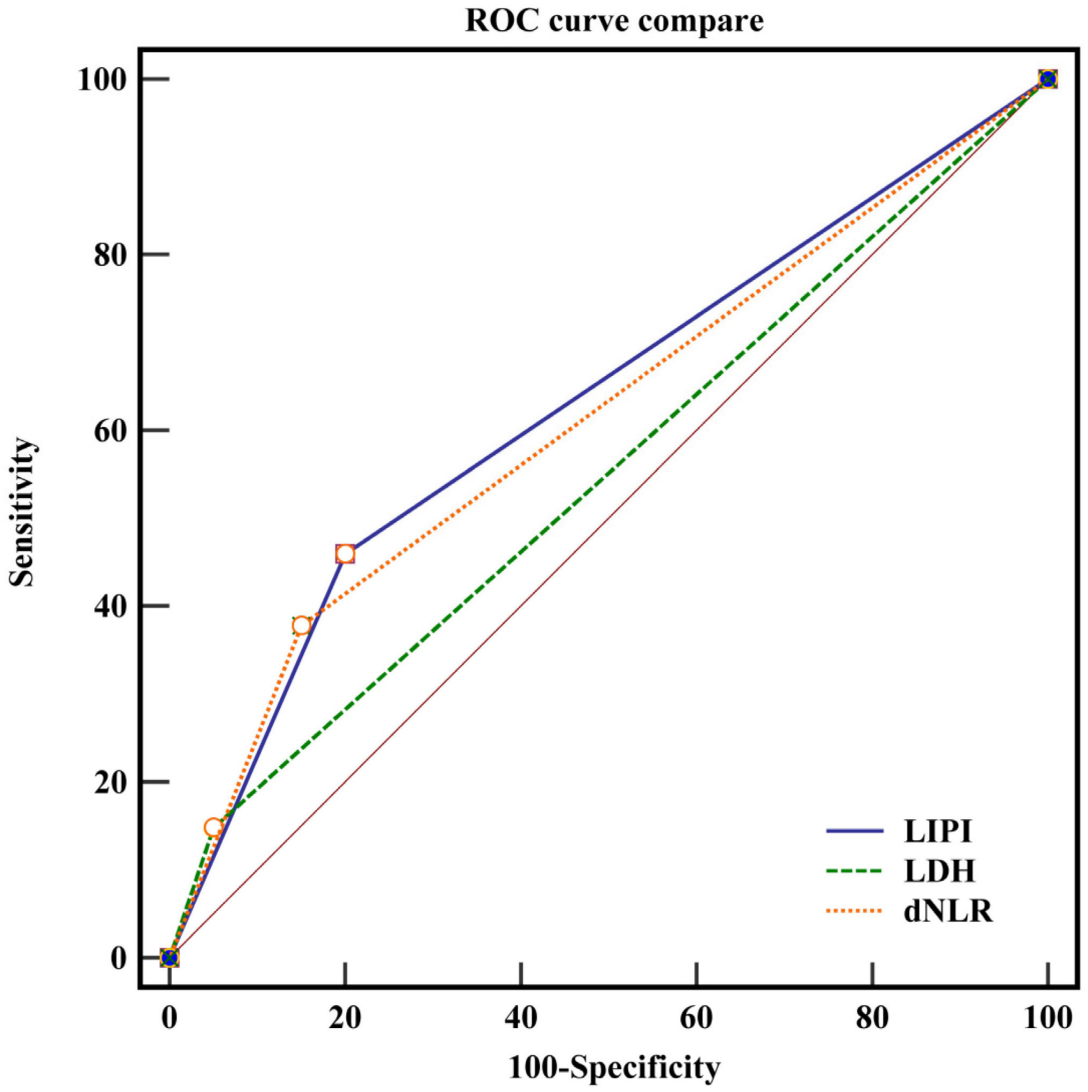
The ROC curve of LIPI, dNLR and LDH.

The median OSs in the good and intermediate/poor LIPI groups were 9.0 (95% CI: 7.351-10.649) and 6.0 months (95% CI: 4.812-7.188), respectively. Univariate analysis revealed that a good LIPI was associated with a higher OS compared to an intermediate/poor LIPI (HR: 0.734; 95% CI: 0.570-0.945; *P* = 0.003; Figure 2). It also revealed that other prognostic factors for OS included male sex (HR: 1.583; *P* < 0.001), smoking history (HR: 1.374; *P* = 0.010), high CEA (HR: 1.182; *P* = 0.005), high CA19-9 (HR: 1.189; *P* = 0.036), chest abdominal effusion (HR: 1.352; *P* = 0.034), and liver metastases (HR: 1.378; *P* = 0.022) (Table 2).

After adjusting for potential confounding factors, patients in the good LIPI group showed higher OSs compared to those in the intermediate/poor LIPI group (HR, 0.720; 95% CI: 0.554-0.935; *P* = 0.014). Moreover, we noted that OS was also affected by male sex (HR: 1.595; 95% CI: 1.143-2.225; *P* = 0.006), high CEA (HR: 1.150; 95% CI: 1.011-1.308; *P* = 0.034), and high CA19-9 (HR: 1.215; 95% CI: 1.016-1.453; *P* = 0.033). Smoking status, chest and abdominal effusion, and liver metastases were not found to be associated with OS after adjusting for confounders (Table 2).

### Subgroup analysis

Subgroup analyses of the role of LIPI in OS were also performed (Table 3). We noted that good LIPI was associated with an improvement in OS compared to intermediate/poor LIPI in patients with no smoking history, no alcohol consumption history, high CEA, high CA19-9, obstructive jaundice, 2-3 organs transferred, liver metastases, and chemotherapy regimens that did not include G. LIPI was not significantly associated with OS in any other subgroups.

## Discussion

The objective of this study was to evaluate the predictive significance of LIPI in relation to OS in patients with PDAC treated with chemotherapy. To ensure the reliability of the dNLR cutoff value, we used X-tile software to calculate it based on time-dependent ROC curves. This method allowed for an accurate assessment of LIPI's impact on PDAC prognosis. We recruited 256 patients with PDAC, and the median OS for the good LIPI group was 9.0 months, compared to 6.0 months for the intermediate/poor LIPI group. Patients in the good LIPI group had a significantly longer OS than those in the intermediate/poor LIPI group. After adjusting for potential confounding factors, other significant prognostic factors included sex, CEA levels, and CA19-9 levels. The prognostic role of LIPI for OS was statistically significant in the subgroups of patients with no smoking history, no alcohol consumption history, high CEA, high CA19-9, obstructive jaundice, 2-3 organ transplantations, liver metastases, and chemotherapy regimens that did not include G. These findings highlight the potential of LIPI as a valuable prognostic tool in predicting OS for PDAC patients undergoing chemotherapy.

A previous study examined 205 patients with PDAC who were treated with radical resection, and assessed the value of preoperative LIPI in predicting OS and recurrence-free survival (RFS) 22. They found that a preoperative intermediate/poor LIPI was associated with poor OS and RFS. Moreover, vascular invasion and chemotherapy were found to affect OS, while RFS was affected by CA-125 level and vascular invasion. However, this study focused on patients diagnosed with stage I-III PDAC, leaving the prognostic value of LIPI for stage IV or locally advanced PDAC unclear. To address this gap, the objective of the current study was to evaluate the prognostic significance of LIPI in terms of OS in patients with locally advanced or metastatic PDAC who are receiving chemotherapy.

We discovered a significant association between the LIPI and OS in PDAC patients undergoing chemotherapy. LIPI is computed based on two key factors: LDH and dNLR. Both LDH and dNLR have well - established links to patient prognosis across various solid cancers, as evidenced by previous studies 23, 24. LDH is a crucial enzyme in tumor cell energy metabolism. In pancreatic cancer, it is highly expressed not only in cancer cells but also in peripheral blood and tissues. This elevated expression is associated with increased tumor invasiveness, which directly impacts the prognosis of pancreatic cancer patients 25, 26. The high levels of LDH may be attributed to the enhanced glycolytic activity of pancreatic cancer cells, a characteristic known as the Warburg effect. This increased glycolysis provides the necessary energy for tumor growth, invasion, and metastasis. Moreover, dNLR is an indicator of the body's internal inflammatory state, which in turn reflects the tumor microenvironment. Neutrophils, a component of the dNLR calculation, can actively influence the tumor microenvironment. They secrete various cytokines and chemokines that promote tumor growth, angiogenesis, and immune evasion. For example, neutrophils can release vascular endothelial growth factor, which stimulates the formation of new blood vessels to supply nutrients to the growing tumor. Additionally, they can interact with tumor cells and other immune cells, modulating the inflammatory response in a way that is favorable for tumor progression 27. Conversely, lymphocytes, the other component of dNLR, play a vital role in anti-tumor immunity. Lymphocyte infiltration into the tumor microenvironment is significantly associated with a better response to immunotherapy and improved prognosis. Cytotoxic T lymphocytes can directly recognize and kill tumor cells, while helper T cells can secrete cytokines that enhance the immune response. Regulatory T cells, although having an immunosuppressive function, can also be balanced by the presence of effector T cells and other immune cells in a healthy immune microenvironment 28. Given the complexity of PDAC biology, a single biomarker may not comprehensively and accurately reflect the prognosis. By combining dNLR and LDH in the form of LIPI, we can capture multiple aspects of the tumor microenvironment, including inflammation, energy metabolism, and immune cell balance 29. This integrated approach provides a more reliable and comprehensive prognostic tool for PDAC patients.

We also performed an exploratory analysis according to patients' characteristics, which revealed that the association between LIPI and OS in patients with PDAC could be affected by smoking status, alcohol intake, CEA level, CA19-9 level, obstructive jaundice, number of transplanted organs, liver metastases, and chemotherapy regimens. Cigarette smoking is significantly associated with the risk and prognosis of PDAC, which can be explained by the direct effect of cigarette smoke on the tumor cell microenvironment 30. Moreover, the TGF-β pathway in patients with a history of alcohol consumption can lead to the formation of extensive stroma, which can affect the prognosis of PDAC 31. CEA and CA19-9 levels reflect the severity of PDAC and can potentially influence its prognosis 32. The disease status of PDAC is significantly related to obstructive jaundice, number of organ transplants, and liver metastases; whereas chemotherapy regimens are significantly related to OS in patients with locally advanced or metastatic PDAC. These findings suggest that while LIPI is a valuable prognostic tool, its predictive power for OS in PDAC patients can be modulated by these additional clinical and biological factors.

This study had several key limitations worth noting. First, the retrospective nature of this analysis is a significant constraint. Retrospective cohort studies inherently carry the risk of recall and selection biases. These biases can distort the results and may limit the generalizability of the findings. Given that our data was collected from past medical records, there could be missing or inaccurate information, and the selection of patients for the study may not be entirely representative of the broader PDAC patient population. Second, the relatively small sample size of 256 patients is another limitation. A larger sample size is generally required to increase the statistical power of the study and to ensure that the results are more robust and applicable to different patient populations. With a small sample, there is a higher chance of random errors and the inability to detect rare events or subtle associations accurately. Third, the effectiveness of currently available chemotherapies for advanced PDAC is modest, and the choice of chemotherapy regimen is often at the discretion of the treating physician. This variability in treatment selection may have introduced additional bias into the study. Fourth, the study primarily focused on the role of LIPI in predicting OS; other results regarding disease progression were not investigated. This narrow focus limits the comprehensive understanding of LIPI's prognostic value in PDAC.

Despite these limitations, we have demonstrated that a good LIPI is significantly related to longer OS in patients with locally advanced or metastatic PDAC undergoing chemotherapy, particularly in the subgroups of those with no smoking history, no alcohol consumption history, high CEA levels, high CA19-9 levels, obstructive jaundice, 2-3 organs transferred, liver metastases, and chemotherapy regimens that did not include G. Considering the data in this study were sourced from a single center, future studies involving multi - center data collection and analysis are essential to validate our results across different patient populations and healthcare settings. Future research should also aim to increase the sample size, reduce biases associated with treatment selection, and explore other aspects of disease progression in relation to LIPI. This will help to establish the true prognostic potential of LIPI in PDAC more comprehensively.

## Figures and Tables

**Table 1:** The baseline characteristics of enrolled patients

Variable	LIPI group		
	Good (n=154)	Intermediate/poor (n=102)	*P* value
Age (years)	56.11±9.02	54.31±9.11	0.122
Sex			0.867
Male	92 (59.7%)	62 (60.8%)	
Female	62 (40.3%)	40 (39.2%)	
Smoking status			0.664
Yes	60 (39.0%)	37 (36.3%)	
No	94 (61.0%)	65 (63.7%)	
Alcohol intake			0.236
Yes	58 (37.7%)	46 (45.1%)	
No	96 (62.3%)	56 (54.9%)	
CEA			0.006
High (>5 μg/L)	76 (49.4%)	68 (66.7%)	
Normal (0-5 μg/L)	78 (50.6%)	34 (33.3%)	
LDH			< 0.001
High (>250 U/L)	0 (0.0%)	32 (31.4%)	
Normal (0-250 U/L)	154 (100.0%)	70 (68.6%)	
dNLR			< 0.001
> 2.3	0 (0.0%)	83 (81.4%)	
≤ 2.3	154 (100.0%)	19 (18.6%)	
PLT (*10^9^/L)	197.5 (138.2-249.8)	206.5 (151.7-266.2)	0.430
ALB (U/L)	40.7 (38.5-43.8)	40.2 (36.0-43.3)	0.072
Diabetes			0.585
Yes	33 (21.4%)	19 (18.6%)	
No	121 (78.6%)	83 (81.4%)	
Obstructive jaundice			0.003
Yes	43 (28.3%)	13 (12.7%)	
No	109 (71.7%)	89 (87.3%)	
Chest abdominal Effusion			0.041
Yes	28 (18.4%)	30 (29.4%)	
No	124 (81.6%)	72 (70.6%)	
Number of organs transferred			0.019
2-3	23 (14.9%)	31 (30.4%)	
1	100 (64.9%)	59 (57.8%)	
0	31 (20.1%)	12 (11.8%)	
Liver metastases			0.343
Yes	114 (74.0%)	80 (79.2%)	
No	40 (26.0%)	21 (20.8%)	
Chemotherapy regimens			0.410
Included G	41 (26.6%)	32 (31.4%)	
Others	113 (73.4%)	70 (68.6%)	

**Table 2:** Univariate and multivariate analyses of prognostic factors for overall survival

Variable	Univariate		Multivariate	
	HR (95% CI)	P value	HR (95% CI)	P value
Age	1.007 (0.993-1.022)	0.316		
Sex				
Male	1.583 (1.226-2.046)	< 0.001	1.595 (1.143-2.225)	0.006
Female	Ref		Ref	
Smoking status				
Yes	1.374 (1.063-1.777)	0.010	1.041 (0.752-1.442)	0.807
No	Ref		Ref	
Alcohol intake				
Yes	1.152 (0.896-1.482)	0.243		
No	Ref			
LIPI				
Good	0.734 (0.570-0.945)	0.003	0.720 (0.554-0.935)	0.014
Intermediate/poor	Ref		Ref	
CEA				
High	1.182 (1.043-1.341)	0.005	1.150 (1.011-1.308)	0.034
Normal	Ref		Ref	
CA19-9				
High	1.189 (1.001-1.411)	0.036	1.215 (1.016-1.453)	0.033
Normal	Ref		Ref	
PLT	2.372 (0.288-19.569)	0.422		
ALB	0.851 (0.568-1.276)	0.436		
Diabetes				
Yes	0.966 (0.712-1.311)	0.813		
No	Ref			
Obstructive jaundice				
Yes	1.118 (0.829-1.508)	0.439		
No	Ref			
Chest abdominal effusion				
Yes	1.352 (1.004-1.821)	0.034	1.147 (0.841-1.563)	0.386
No	Ref		Ref	
Number of organs transferred				
3	0.548 (0.246-1.220)	0.141		
2	0.694 (0.325-1.485)	0.347		
1	0.486 (0.217-1.088)	0.079		
0	Ref			
Liver metastases				
Yes	1.378 (1.029-1.845)	0.022	1.116 (0.821-1.518)	0.482
No	Ref		Ref	
Chemotherapy regimens				
Included G	0.914 (0.696-1.200)	0.493		
Others	Ref			

**Table 3:** Subgroup analyses for the role of LIPI on OS

Variable	Subgroups	Multivariate	
		HR (95% CI)	*P* value
Sex	Male	0.682 (0.459-1.014)	0.058
	Female	0.666 (0.389-1.138)	0.137
Smoking status	Yes	0.788 (0.477-1.304)	0.355
	No	0.616 (0.418-0.906)	0.014
Alcohol intake	Yes	1.036 (0.627-1.709)	0.891
	No	0.572 (0.381-0.857)	0.007
CEA	High	0.655 (0.447-0.959)	0.030
	Normal	0.848 (0.517-1.392)	0.515
CA19-9	High	0.588 (0.428-0.807)	0.001
	Normal	1.297 (0.547-3.073)	0.555
Diabetes	Yes	0.481 (0.231-1.003)	0.051
	No	0.805 (0.577-1.122)	0.200
Obstructive jaundice	Yes	0.399 (0.178-0.894)	0.026
	No	0.768 (0.556-1.061)	0.110
Chest abdominal Effusion	Yes	0.813 (0.401-1.651)	0.567
	No	0.724 (0.518-1.013)	0.060
Number of organs transferred	2-3	0.408 (0.200-0.829)	0.013
	0-1	0.798 (0.564-1.130)	0.203
Liver metastases	Yes	0.709 (0.506-0.995)	0.046
	No	0.662 (0.332-1.321)	0.242
Chemotherapy regimens	Included G	0.739 (0.419-1.302)	0.295
	Others	0.690 (0.477-0.998)	0.049

## Abbreviations

CA19-9: carbohydrate antigen 19-9
CEA: carcinoembryonic antigen
CIs: confidence intervals
dNLR: derived neutrophil-to-lymphocyte ratio
HRs: hazard ratios
LDH: lactate dehydrogenase
LIPI: lung immune prognostic index
OS: overall survival
PDAC: pancreatic ductal adenocarcinoma
RFS: recurrence-free survival
ROC: receiving operator characteristic
